# Very high prevalence of infection with the human T cell leukaemia virus type 1c in remote Australian Aboriginal communities: Results of a large cross-sectional community survey

**DOI:** 10.1371/journal.pntd.0009915

**Published:** 2021-12-08

**Authors:** Lloyd Einsiedel, Hai Pham, Mohammad Radwanur Talukder, Kerry Taylor, Kim Wilson, John Kaldor, Antoine Gessain, Richard Woodman

**Affiliations:** 1 Baker Heart and Diabetes Institute, Alice Springs, Northern Territory, Australia; 2 Poche Centre for Indigenous Health and Wellbeing, Alice Springs, Northern Territory, Australia; 3 National Serology Reference Laboratory, Melbourne, Australia; 4 Kirby Institute, University of New South Wales, Sydney, Australia; 5 Institut Pasteur, Paris, France; 6 Flinders University, Adelaide, Australia; Kumamoto University: Kumamoto Daigaku, JAPAN

## Abstract

Infection with the human T cell leukaemia virus type 1 (HTLV-1) subtype C is endemic among Aboriginal people in central Australia. To provide insights into the risk factors for transmission, we conducted the first large-scale, community-based prevalence study in seven remote Aboriginal communities. Residents >2 years old were invited to participate in the study between August 2014 and June 2018. HTLV-1 infection was defined as a positive western blot (WB) test or a positive HTLV-1 PCR. 720 community residents participated in the study (children <15 years, 142; adults, 578). Prevalences for children and adults were 3.5% (5/142) and 36.8% (213/578), respectively, reaching 49.3% (106/215) for those older than 45 years. A wide range of proviral loads were measured for both asymptomatic and symptomatic participants with no difference within groups according to age or gender; however, median PVL was 1.34 log10 higher for symptomatic participants. The adult prevalence of HTLV-1 infection in central Australia is the highest reported worldwide. Sexual contact is likely to be the predominant mode of transmission.

## Author summary

In the first large scale community study of HTLV-1 prevalence in central Australia we found an adult prevalence of 36.8%, the highest reported worldwide. Prevalence increased with age suggesting that sexual contact may be the predominant mode of transmission. Although a wide range of HTLV-1 proviral loads was found, in nearly 40% of participants this exceeded 1000 copies per 10^5^ peripheral blood leukocytes, a level that has been associated with an increased risk of chronic lung disease and death in this population. The median PVL for participants who were symptomatic with HTLV-1 associated inflammatory diseases was 1.34 log10 higher than that of those who were asymptomatic. These data demand a public health initiative to reduce HTLV-1 transmission among Aboriginal Australians.

## Introduction

The human T cell leukaemia virus type 1 (HTLV-1) is a human retrovirus that causes a chronic infection, which preferentially involves CD4+ T cells [1]. Worldwide, HTLV-1 infects an estimated 5–10 million people, predominantly in focal geographic areas of high endemicity in southern Japan, the Caribbean basin, parts of South America and inter-tropical Africa [2]. An endemic focus is also present in central Australia where infection is with the ‘Australo-Melanesian’ HTLV-1 subtype C [2]. Transmission of HTLV-1 is via exposure to infected lymphocytes through blood contact, condomless sexual intercourse and breast-feeding [1]. Sexual transmission is thought to be the major mode of transmission in most endemic areas [3–5], including in Japan where mother-to-child transmission (MTCT) has fallen markedly in response to routine antenatal screening followed by counselling to limit or avoid breast-feeding by mothers with HTLV-1 [6].

A minority of people with HTLV-1 develop serious HTLV-1 associated complications including a rapidly progressive haematological malignancy (adult T cell leukaemia/lymphoma, ATL) [7,8] and several inflammatory disorders, the best studied of which is HTLV-1 associated myelopathy (HAM) [9,10]. Risk of these conditions is associated with a higher number of HTLV-1 infected cells in peripheral blood (HTLV-1 proviral load, PVL) [1], which was also a risk factor for pulmonary disease and gait abnormalities in a recent clinical survey in central Australia [11].

Notwithstanding recent international interest in the high prevalence of HTLV-1 infection among Aboriginal adults in central Australia [12], progress in understanding the epidemiology of HTLV-1 in this region has been slow. Infection with HTLV-1 was first reported in central Australia in 1988 [13], and each of the major recognised complications of HTLV-1 infection, including HAM and ATL, have now been reported from that region [14]. Nevertheless, actual community-based prevalence data are only available for a single small community in which 40% of adults were infected [15]. Consequently, inferences about prevalence and likely modes of transmission are derived from hospital-based [14,16] and laboratory testing series [17]. These studies have shown high seropositivity rates among Aboriginal adults and a far higher HTLV-1 seropositivity in central Australia compared to areas that are further north [17], suggesting that areas of high endemicity are concentrated in the arid interior of the country.

There has so far been no public health strategy to control HTLV-1 transmission in Australian Aboriginal communities. HTLV-1 testing is not included in routine antenatal screening [18], nor did the Australian national collaborative forum on HTLV-1 in 2018 recommend widespread HTLV-1 testing [19]. A better understanding of the patterns of occurrence of HTLV-1 at a community level is critical to guide the development of public health initiatives to reduce transmission [20]. The ethical collection of such data in a vast region that is sparsely populated by people with limited health literacy presents a considerable challenge. We now report the first large-scale, community-based, survey of HTLV-1c seroprevalence across a large area of central Australia, using a research method developed in partnership with participating communities. The study also systematically measured HTLV-1 PVL, providing an insight into relationships between HTLV-1 PVL, age and gender in this community setting.

## Methods

### Ethics statement

The study was developed in collaboration with Aboriginal stakeholders and primary health care providers and approved by the Central Australian Human Research Ethics Committee (HREC-14-242, HREC 15–322 and HREC 16–384). Informed written consent was obtained from all adult participants and, in the case of children, from their legal guardians.

### Study setting

**Fig 1:**
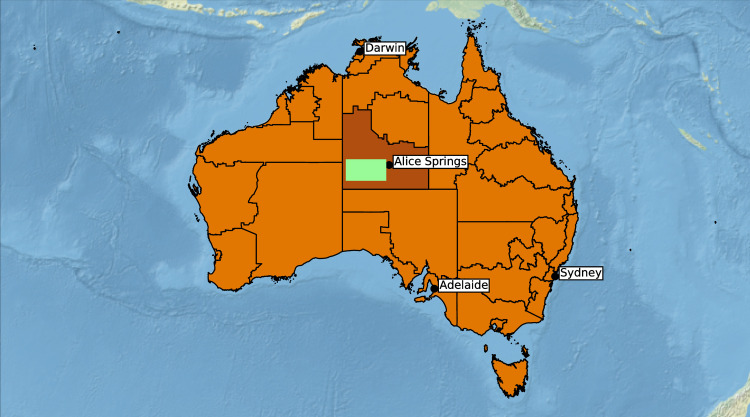
Map of Australia showing the study area of approximately 68,000 km^2^ (green rectangle) within the Apatula Indigenous region (dark brown). The basemap layer was obtained from Natural Earth: https://www.naturalearthdata.com/downloads/50m-natural-earth-2/50m-natural-earth-ii-with-shaded-relief-and-water/.

Central Australia comprises an area of some 1,000,000 km^2^ in which most Aboriginal Australians reside in isolated communities under conditions of considerable socio-economic disadvantage. Most communities have populations of only several hundred people who often speak English as their second or third language. Infrastructure is limited but generally includes a small nurse-staffed, primary health care (PHC) clinic. The region is served by a single, well resourced, 186-bed hospital (Alice Springs hospital, ASH) in Alice Springs, which is the only town in the region. Seven remote communities (Fig 1) (estimated resident population (Australian census, 2016): children (<15 years), 296; adults (≥15 years), 1241) [21] participated in the study, during visits by the research team to the communities between 25 August 2014 and 30 June 2018.

For each community, the project was first discussed with community leaders, and then proposed to the community after ethics approval was granted. All Aboriginal community members >2 years old were eligible for recruitment, unless unable to give informed written consent. Consent for children was obtained from their legal guardians. Information about the project, and for consent, was provided in primary languages [15]. Summary data from one community have been presented previously [15].

A central survey site, which was generally the PHC clinic, was established in each community with the assistance of community members. Aboriginal researchers, fluent in local Aboriginal languages, provided transport to the survey site as needed, facilitating active recruitment and controlling participant numbers.

In order to compare PVL between asymptomatic and symptomatic participants, health records held at local clinics and at ASH were reviewed within several months of recruitment by project managers (HP, RT) who were blinded to HTLV-1 status. Records for five years prior to recruitment were reviewed, demographic information was collected and details of HTLV-1 associated conditions were recorded without regard to the date of diagnosis. Results of all chest imaging and neuroimaging from five years prior to recruitment to 30 November 2018 were reviewed at ASH (LE) blind to HTLV-1 status. The symptomatic group comprised predefined HTLV-1 related diseases: i) a haematological diagnosis of ATL, ii) radiologically defined bronchiectasis/bronchiolitis, iii) non-diabetic eye diseases diagnosed by an ophthalmologist, and documentation in the medical records of iv) myositis, v) infective dermatitis and vi) a diagnosis of ‘myelopathy’ or neurological symptoms associated with HTLV-1 [22,23] that were not explained by other causes. Clinical details for a subset of participants who were examined by a specialist in internal medicine (LE) have been published previously [11].

Whole blood was collected in EDTA tubes from adults and older children by venepuncture. Peripheral blood buffy coats (PBBC) and plasma were recovered and the samples stored at ASH at -80°C until testing. Collection of blood on Whatman 903 cards by finger prick using a lancet (dried blood spots, DBS) was offered to children less than approximately 10 years.

### HTLV-1 serologic and molecular markers

Buffy coats and DBS were shipped to the National Serology Reference Laboratory, Melbourne. HTLV serology screening was performed on samples collected by venepuncture (662 participants) using an enzyme immune-assay (Murex HTLV I + II, DiaSorin Dartford, UK) and a particle agglutination assay (Serodia HTLV-1, Fujirebio, Tokyo, Japan). All samples reactive on either screening assay were tested by Western blot (HTLV-I/II Blot2.4, MP Diagnostics, Singapore), with the result classified as positive, indeterminate or negative according to manufacturer’s criteria.

HTLV-1c PVL was assessed for all participants by real-time polymerase chain reaction (PCR) using DNA extracted from PBBC for 662 participants as previously described [15], and from DBS for 58 young children. Four 6mm discs were punched from the DBS for each participant, combined in one tube, eluted and DNA extracted using a QIAamp DNA Blood Mini Kit according to the manufacturer’s instructions. For all participants, the number of copies of HTLV-1 per peripheral blood leukocytes (PBL) was then calculated and the proviral load expressed as HTLV-1 copies per 1 x 10^5^ PBL. The lower limit of detection for PBBC samples was 6.5 copies for HTLV-1 (95% CI 5.4–8.4) and 15.6 copies for albumin (95% CI 12.9–20.0).

HTLV-1 infection was defined as a positive WB test or a positive HTLV-1 PCR test. Sufficient blood for serology was not available for the 58 young children from whom only DBS samples were collected and in these cases HTLV-1 infection was defined as a positive PCR test.

### Statistical analysis

All analysis was performed using the Stata statistical software version 15.1 (StataCorp, USA). The primary outcome was HTLV-1 infection, as defined above. Categorical data were described using frequencies and percentages and continuous data using mean and standard deviation. Differences between group means were assessed using independent t-tests and chi-squared tests of Fishers Exact test as appropriate. Logistic regression was used to determine predictors of binary outcomes with variables significant at p<0.20 in univariate analysis considered for inclusion in multivariate analysis. Associations between quantities measured within the same individual were assessed using the Pearson r correlation coefficient. A 2-sided Type 1 error rate of alpha = 0.05 was used for assessing statistical significance.

## Results

During the study period, 720 community residents participated in the study (children <15, 142; adults, 578), representing 46.8% of the estimated resident population of the communities (children, 48.0%; adults 46.6%). The age and gender profile of participants closely resembled that of the overall resident population (S1 Table).

**Fig 2:**
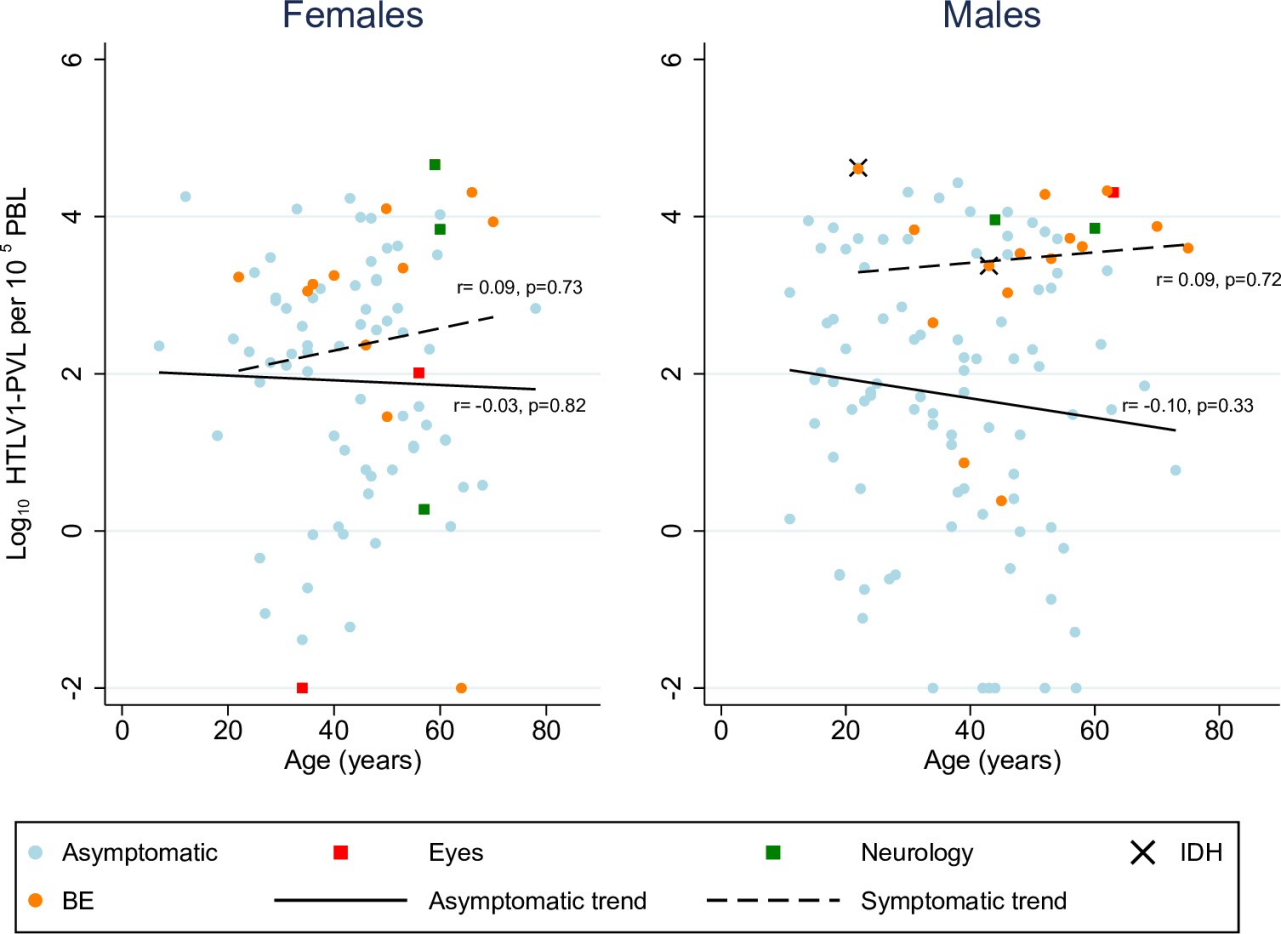
HTLV-1c proviral loads for 91 (asymptomatic, 75; symptomatic, 16) female and 128 (asymptomatic, 109; symptomatic, 19) male participants. Pearson correlation coefficients and p-values for PVL versus age are shown for symptomatic and asymptomatic subjects (symptomatic group, solid lines; asymptomatic group, dashed lines). The symptomatic group includes i) radiologically defined bronchiectasis/bronchiolitis (27)(orange circles), ii) uveitis (2), retinitis (1) or chronic bilateral corneal opacities (1) (red squares), iii) myositis (1) (green square), iv) infective dermatitis (black cross)(2) and v) neurological symptoms associated with HTLV-1 (5)(green squares). Three participants had more than one HTLV-1 associated condition: a 59 year old woman (PVL 4.65 log10 copies per 10^5^ peripheral blood leukocytes, PBL) had possible HAM and eye disease (green square); a 58 year old man (PVL 3.62 log10 copies per 10^5^ PBL) had possible HAM and bronchiectasis (orange circle); a 43 year old man (PVL 3.37 log10 copies per 10^5^ PBL) had bronchiectasis and infective dermatitis (orange circle with black cross). Median (IQR) HTLV-1 PVL log10 HTLV-1 copies per 10^5^ PBL amongst females were 2.31 (1.06–3.14) for asymptomatic females (n = 75) and 3.19 (1.73–3.89) for symptomatic females (n = 16) (p = 0.120). Median (IQR) HTLV-1 PVL log10 HTLV-1 copies per 10^5^ PBL amongst males were 2.02 (0.68–3.27) for asymptomatic males (n = 109) and 3.72 (3.37, 4.28) for symptomatic males (n = 19) (p<0.001). **Abbreviations**: BE; radiologically defined bronchiolitis/bronchiectasis; eyes, non-diabetic eye diseases; HTLV-1, human T cell leukaemia virus; IDH, infective dermatitis associated with HTLV-1; neurology, ‘myelopathy’ or neurological symptoms associated with HTLV-1; p, p value; PBL, peripheral blood leukocytes; r, Pearson correlation coefficient between PVL and age by gender.

HTLV-1 infection was confirmed for 218 participants on the basis of WB or PCR positivity (WB positive/PCR positive, 209; indeterminate WB/PCR positive, 8; DBS/positive PCR, 1). There was a wide range in HTLV-1 PVL (Fig 2), but no significant difference was found between females (median PVL, 230; interquartile range (IQR) 12.0 to 1780 copies per 10^5^ PBL) and males (median HTLV-1 PVL, 205; IQR 10.6 to 3630 copies per 10^5^ PBL)(p = 0.98). There was no relationship between HTLV-1 PVL and age for females or males (Fig 2), and this was also true when symptomatic participants were excluded from the analysis (Fig 2).

Adults with HTLV-1 were more likely than participants without HTLV-1 to have conditions that were HTLV-1 associated (bronchiolitis/bronchiectasis, 27/39; possible HAM, 5/5; non-diabetic eye diseases, 4/4; infective dermatitis, 2/2; myositis, 1/1). Among 35 participants who met the definition of symptomatic HTLV-1 infection, 38 HTLV-1 associated conditions were recorded including radiologically confirmed bronchiectasis/bronchiolitis (27), possible HAM (5) (one with bronchiectasis, one with chronic bilateral corneal opacities), infective dermatitis (2) (one with bronchiectasis), non-diabetic eye diseases (4)(uveitis, 2; retinitis, 1, chronic bilateral corneal opacities, 1) and myositis (1). Evidence of neurological disease included i) gait abnormalities (three participants: a 59 year old woman with corneal opacities and urinary incontinence, PVL 45614 copies per 105 PBL; a 58 year old man with bronchiectasis and bladder dysfunction for whom a diagnosis of ‘myelopathy’ was also recorded in medical records, PVL 4160 copies per 10^5^ PBL; a 57 year old woman for whom a diagnosis of ‘myelopathy’ was also recorded in medical records, PVL 1.88 copies per 10^5^ PBL); ii) upper limb weakness, neuropathic pain, back pain and dysphagia in a 60 year old woman (PVL 6888 copies per 10^5^ PBL) and iii) neuropathic pain, with back pain and bilaterally increased lower limb tone in a 44 year old man (PVL, 9100 copies per 10^5^ PBL). Spinal canal stenosis was excluded by CT in four cases; no neuroimaging was done for the 59 year old woman with an abnormal gait and urinary incontinence. In no case was HTLV-1 testing done on cerebrospinal fluid (CSF).

An HTLV-1 PVL above 1000 copies per 10^5^ PBL was recorded for 48 of 127 (37.8%) male participants and 31 of 91 (34.0%) female participants with HTLV-1. Notwithstanding some overlap in the PVL values of symptomatic and asymptomatic participants (Fig 2), the median HTLV-1 PVL was significantly higher for those who were symptomatic (symptomatic, 3.53, (2.65, 3.96); asymptomatic, 2.19 (0.78, 3.16) log10 copies per 10^5^ PBL; p<0.001).

The overall community HTLV-1 prevalence was 30.3% (218/720) (Table 1). Prevalences for children (under 15) and adults (15 and over) were 3.5% (5/142) and 36.8% (213/578), respectively (Table 1). None of the nineteen children aged less than 5 years had HTLV-1, and prevalence remained low for children 5–14 years (4.1%; 5/123). Thereafter, rates increased markedly, reaching 25.0% between 15–24 years and 49.3% (106/215) for adults older than 45 years (Table 1).

**Table 1:** HTLV-1 prevalence by age and gender in seven remote central Australian communities, 2014–18.

	Female				Male				Total			
Age	HTLV-1 positive				HTLV-1 positive				HTLV-1 positive			
	n	(%)	OR (95% CI)^1^	p value	n	(%)	OR (95% CI)^1^	p value	n	(%)	OR (95% CI)^1^	p value
**<15**	3/70	4.3	ref		2/72	2.8	ref		5/142^2^	3.5	ref	
**15–24**	4/50	8.0	1.9 (0.4–9.1)	0.399	25/67	37.3	20.8 (4.7–92.4)	<0.001	29/117	24.8	9.0 (3.4–24.2)	<0.001
**25–34**	16/68	23.5	6.9 (1.9–24.8)	0.003	17/62	27.4	13.2 (2.9–60.0)	0.001	33/130	25.4	9.3 (3.5–24.7)	<0.001
**35–44**	19/64	29.7	9.4 (2.6–33.7)	0.001	26/52	50.0	35.0 (7.7–157.9)	<0.001	45/116	38.8	17.4 (6.6–45.7)	<0.001
**45–54**	26/75	34.7	11.8 (3.4–31.4)	<0.001	35/49	71.4	87.5 (18.8–406.6)	<0.001	61/124	49.2	26.5 (10.2–69.2)	<0.001
**55–64**	16/38	42.1	16.2 (4.3–61.0)	<0.001	13/20	65.0	65.0 (12.1–348.5)	<0.001	29/58	50.0	27.4 (9.8–76.8)	<0.001
**>64**	7/21	33.3	11.2 (2.6–48.6)	0.001	9/12	75.0	105.0 (15.4–715.5)	<0.001	16/33	48.5	25.8 (8.4–79.3)	<0.001
**Total**	91/386	23.6			127/334	38.0	2.0 (1.4–2.7)	<0.001^3^	218/720	30.3		

HTLV-1 infection was confirmed for 218 participants, either on the basis of WB or PCR positivity (WB positive/PCR positive, 209; indeterminate WB/PCR positive, 8; tested by dried blood spot, PCR positive, 1).

1. Unadjusted odds ratio for HTLV-1 status for male and female, and total by age groups.

2. Samples collected by dried blood spots (1/58) and PBBC (4/86).

3. Odds ratio for males relative to females

**Fig 3:**
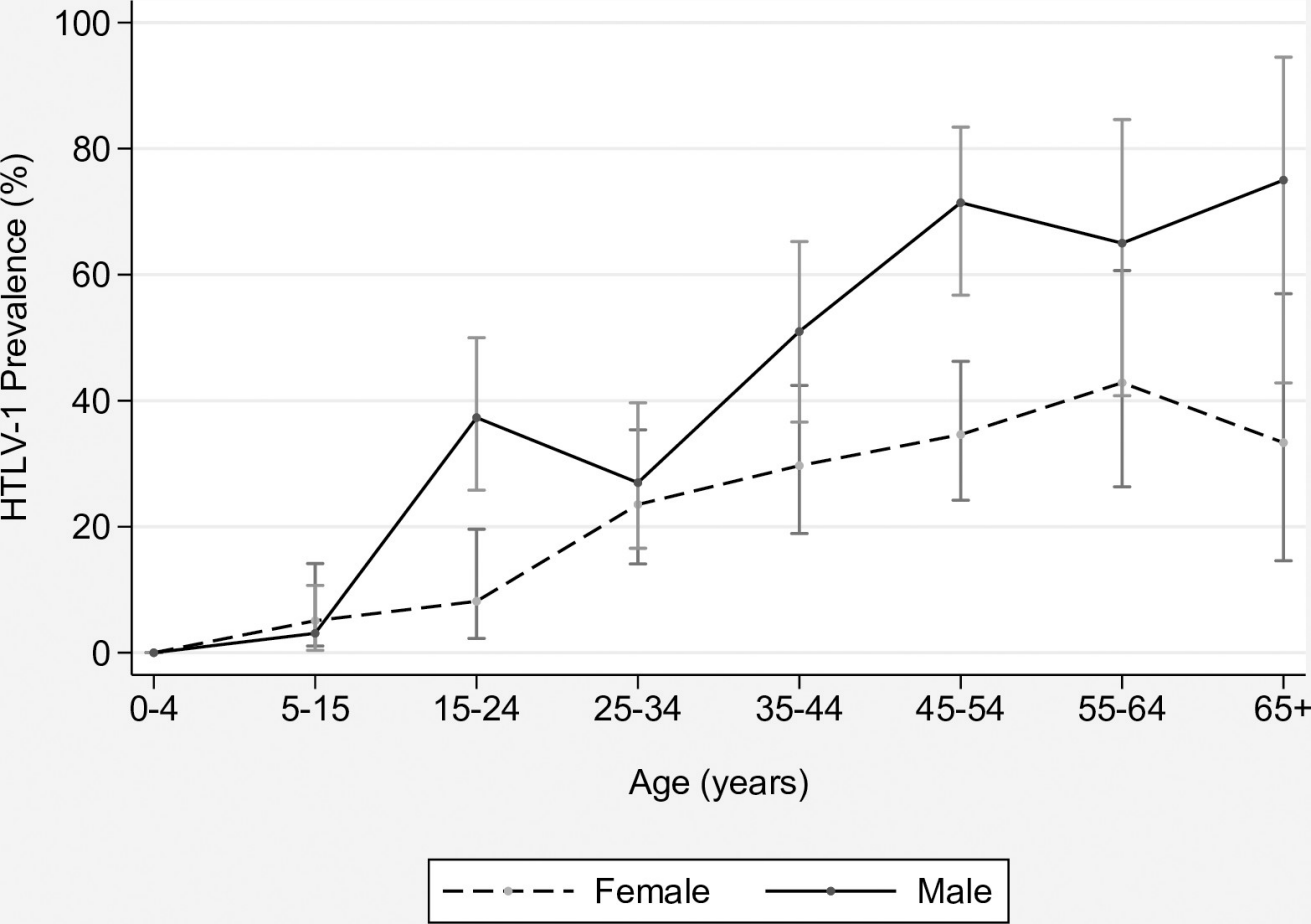
HTLV-1 prevalence according to age for 386 female and 334 male community residents. HTLV-1 infection was confirmed for 218 participants, using HTLV-1 western blots (WB) and/or HTLV-1 PCR (WB positive/PCR positive, 209; indeterminate WB/PCR positive, 8; tested by dried blood spot, PCR positive, 1).

As shown in Fig 3, prevalence was higher for men than women, at 38.0% (127/334) versus 23.6% (91/386); p<0.001). Rates increased sharply for males between age groups 5–14 (4.1%) and 15–24 (37.3%) and continued to increase with age, exceeding 70% for men older than 45 years (Table 1). A more gradual increase in prevalence was apparent for women, which was most marked between age groups 15–24 (8.0%) and 25–34 years (23.5%). Thereafter, prevalence increased to 36.6% for women older than 45 years. Among women of reproductive age (15–39 years), prevalence was 20.0% (32/160).

Adult prevalence was consistently high for all seven communities, ranging from 27% to 48%. In a multivariate model that included age and gender, the odds of HTLV-1 infection increased 4% for each year of age (aOR, 1.04; 95% CI, 1.02, 1.05; p<0.001) and was nearly three times higher for males compared to females (aOR, 2.95; 95% CI, 2.04, 4.27; p<0.001).

Amongst participants aged 14 to 90 years old the incidence rates of HTLV-1 infection were 27.3 (22.2, 33.7) and 15.8 (10.1, 24.9) and for males and females, respectively (IRR = 1.72; 95% CI = 1.31, 2.27; p<0.001). Amongst participants aged 20 to 24 years old the incidence rates were 10.7 (3.5, 33.2) and 2.5 (0.2, 25.1) for males and females, respectively (IRR = 4.36; 95% CI = 1.25, 15.16; p = 0.021). Incidence rates of HTLV-1 infection (95% CI) for asymptomatic and symptomatic men were 44.4 per 100 persons tested (35.9, 52.8) and 82.6 per 100 persons tested (45.5, 119.8), respectively. Comparable rates for asymptomatic and symptomatic women were 24.8 (19.0, 30.6) and 61.5 (31.4, 91.7), respectively. The incidence rate ratio (95% CI) of HTLV-1 infection was 1.70 (1.30, 2.24)(p<0.001) for men relative to women, and was 2.10 (1.46, 3.02)(p<0.001) for symptomatic relative to asymptomatic participants.

## Discussion

In the first large scale, community-based, HTLV-1 prevalence study in Australia we report an adult (≥15 years) prevalence of 36.8%, the highest community prevalence recorded worldwide [4,24]. Micro-geographic variation in prevalence is a well-recognized feature of HTLV-1 occurrence globally [2]. For example, prior to the introduction of public health interventions that were associated with dramatic reductions in prevalence in Japan [6], rates in the south-west of that country exceeded 30% for adults aged >30 years in some villages, but were less than 10% in others nearby [24]. This was not the case in the present study in which HTLV-1 prevalence was consistently high for all participating communities across a large region of central Australia (Fig 1).

The study also systematically measured HTLV-1 PVL in study participants, extending previously published observations from these communities [11] to reveal a wide range of inter-individual HTLV-1 proviral loads, exceeding 1000 copies per 10^5^ PBL for 36% of participants with HTLV-1. An HTLV-1 PVL exceeding this level has been associated with serious adverse outcomes, including bronchiectasis and death, in hospital-based studies in this population [25,26]. A strong association between high HTLV-1 PVL and chronic pulmonary disease was also found among 415 adult residents of these communities who were examined by a physician blinded to HTLV-1 status [11]. In the present study, which used medical records to identify HTLV-1 associated conditions, the median PVL for the symptomatic group was 1.34 log10 higher than that of asymptomatic participants.

Higher HTLV-1 PVL among men and older participants were found in a large cohort of adults recruited from Japanese medical facilities, most of whom were tested for HTLV-1 during an illness or after a relative was found to have HTLV-1 [27]. An increasing PVL with age has therefore been suggested to contribute to the increased risk of HTLV-1 associated diseases that has been observed among older individuals [27]. In contrast, no correlation was found between HTLV-1 PVL and age for either symptomatic or asymptomatic groups in the present community-based study, nor was there any difference in median values according to gender. These findings are consistent with the results of a community survey in Gabon [28] and with studies of adults who attempted blood donation in Brazil [29], the USA [30] and Japan [31,32]. Prospective studies are necessary to more accurately define the risk of developing disease entities that is associated with a given PVL. However, ensuring that participants are correctly classified at baseline will be extremely difficult, particularly for those with bronchiolitis and early HTLV-1 associated pulmonary disease [33], and neurological disease not fulfilling HAM criteria [22,23].

As is typical of other parts of the world with endemic HTLV-1 infection [4,24,34–37], prevalence increased with age in central Australia. This relationship has previously been reported for Aboriginal people in health-care associated studies [38,39]. For example, HTLV-1 seropositivity rates among Aboriginal children (aged less than 11 years) and adults whose blood was collected for a medical audit at ASH in 1977 were 6.4% and 20.6%, respectively [38]. More recently, HTLV-1 prevalences among in-patients at ASH were 1.3% and 35.9% for children and adults, respectively [16]. In a study of patients from the north of the NT, seropositivity rates among children and adults were 0.0% and 1.65%, respectively [39].

The sharp increase in the prevalence of a chronic infection around adolescence suggests that sexual contact may be playing an important role in transmission, and this is considered to be the explanation for the age-prevalence relationship in other HTLV-1 endemic areas [3,6,40]. The role for sexual intercourse in HTLV-1 transmission has been inferred in international studies based on associations between the prevalence of HTLV-1 infection and higher numbers of sexual partners [3,5,40–43], a longer sexual relationship with a partner at risk of HTLV-1 infection [44], the presence of genital lesions [45] and a history of sexually transmitted infections (STI), including syphilis [5,45] and gonorrhoea [5]. A previous bacterial STI was also a risk factor for HTLV-1 infection in an earlier study at ASH [16]. Genital lesions may directly increase risk of HTLV-1 infection during condomless sexual intercourse by exposing target cells to HTLV-1 infected lymphocytes in body fluids [46]. If so, the high rates of bacterial STIs [27] among young Aboriginal people in the NT might enhance the risk of HTLV-1 transmission.

Among adults, the higher prevalence that we observed in men compared to women differed from international reports, which have found a higher prevalence in women [4,24]. In the present study, we were unable to collect sensitive information relating to sexual behavior or other possible risk factors for HTLV-1 transmission, and the cause for this gender-based difference is unclear. Although condomless sexual intercourse is considered to be the major mode of transmission worldwide [3,6,40], sharing devices used for self-flagellation in a religious context was associated with HTLV-1 transmission in a recent report from the UK [47], which suggests that predisposing factors may vary in different populations. Further studies by Aboriginal researchers are needed to more clearly define the risk factors for HTLV-1 transmission in this population.

Research from Japan and other endemic countries has shown that early childhood infection is strongly associated with a longer duration of breast-feeding. This increased risk is thought to result from the continued exposure of infants to HTLV-1 infected cells in breast milk after the loss of protective maternal antibodies [35], which occurs within 12 months of age [48]. Infection rates therefore increase substantially with the duration of breast-feeding; 3.9–9.0% at 6–7 months [35,48], 20.3% after 6 months [35] and 32% if breast-feeding is continued longer than 12 months [49]. The overall childhood prevalence of 3.5% that was recorded by us is consistent with that which would result from a 20% mother-to-child transmission rate and a 20% prevalence of infection among women aged 15–39 years. Although this childhood prevalence rate is somewhat higher than that reported in other marginalised populations [4], only one positive test was recorded for a child aged less than ten years and further studies are required to determine the true rates of mother to child transmission in this region.

Strengths of this study include its community setting and the blinding of participants and researchers to HTLV-1 status until after medical records were reviewed. Nevertheless, a number of limitations must be acknowledged. First, based on ABS population data, less than 50% of the estimated resident population was recruited, raising the possibility of selection bias. We are also unable to calculate a regional HTLV-1 prevalence because only seven communities participated in the study. However, we previously reported an overall adult prevalence of 35.9% for adults admitted to ASH from across the region [16] and rates are even higher among patients from adjacent areas of the states of South Australia and Western Australia [14]. The HTLV-1 endemic area may therefore extend across a vast area of Australia that includes parts of Western Australia, South Australia and the Northern Territory. Second, HTLV-1 PCR applied to DBS samples has a lower sensitivity than it does for PBBC samples. The use of DBS samples to determine HTLV-1 status for young children may therefore underestimate HTLV-1 prevalence in this group. However, the similarly low prevalence based on PBBC specimens in the larger group of children aged 10–15 years in our survey (4.7%), and the consistency with seropositivity rates recorded for children who were in-patients at ASH in 1977 (6.4% [38]) and 2016 (1.3% [16]), gives confidence in the validity of the results. Despite the low prevalence, acquisition of HTLV-1 infection in early life is clinically very important because of its strong association with ATL, a fatal haematological malignancy that has been reported at ASH [50]. Finally, the categorization of participants as symptomatic or asymptomatic according to diagnoses recorded in their medical records is likely to underestimate the prevalence of HTLV-1 associated diseases, and this limits our interpretation of comparisons of PVL data between these groups. For example, chest HRCT are routinely requested to further characterize abnormalities apparent on chest x-ray, the sensitivity of which is poor for the diagnosis of bronchiolitis and early bronchiectasis [51]. Consequently, chest x-rays were the only imaging modality for most participants, and 21 participants with HTLV-1 had no chest imaging done during the study period. Similarly, possible HTLV-1 associated neurology was recorded in medical records for only five of 578 (0.86%) adults in the present study compared to 15 of 415 (3.6%) adults who were identified by clinical examination [11]. In the present study, the clinical description was consistent with HAM in three cases, and an association with HTLV-1 was made more likely for two of these participants by the very high PVL and the presence of other HTLV-1 associated conditions (bronchiectasis and eye disease). Nevertheless, in no case were we able to confirm this diagnosis using medical records because these were not written with HTLV-1 associated diseases in mind, HTLV-1 testing of CSF was not done and other possible causes, such as tertiary syphilis and vitamin B12 deficiency, were not excluded. This could lead to misclassification that might affect the true difference in PVL between symptomatic and asymptomatic groups, but this is unlikely to alter our conclusion that HTLV-1 PVL did not vary with age or gender for either group. Finally, PVL were measured using DNA extracted from PBBC and the differential leukocyte count was not available for many participants in our remote study setting. HTLV-1 PVL data are therefore presented relative to the number of peripheral blood leukocytes and our data cannot be compared to that from other studies that have used peripheral blood mononuclear cells as their reference. Nevertheless, our methods have strong internal validity that has allowed clinically meaningful comparisons between symptomatic and asymptomatic participants in previous studies [11,25,26].

In the first large-scale, community-based, epidemiological study of HTLV-1 in Australia, we recorded an adult HTLV-1 prevalence of 36.8%. Based on age-specific prevalence, sexual contact appears likely to be the major mode of transmission in this region, as it is in other endemic countries [4,6]. Each of the recognised complications of HTLV-1 has been described in central Australia [14]. Infection with HTLV-1 is also associated with an increased risk of death in all endemic areas where mortality has been studied [52], and a higher HTLV-1 PVL predicts death at ASH [25,26].

Prevention is particularly important because HTLV-1 infection is lifelong and there is no treatment that directly targets the virus. Results of the present study argue for the implementation of a coordinated program to inform Aboriginal Australians of the risks posed by HTLV-1 infection and to reduce the risk of viral transmission in this population. In Japan, rising incidence rates among young adults in non-endemic, metropolitan centres have followed their migration from HTLV-1 endemic regional areas [4]. A recent report of infective dermatitis in an Aboriginal girl born in a major Australian city [53] suggests that increasing population mobility may similarly make HTLV-1 infection more relevant to Australian settings beyond the remote communities of central Australia.

## Supporting information

S1 Table Estimated resident population data not available for children aged 3–4 years (n = 18). No data for gender available for two communities (n = 102).
(DOCX)
