# Supplementary material for: Very high prevalence of infection with the human T cell leukaemia virus type 1c in remote Australian Aboriginal communities: Results of a large cross-sectional community survey
Source: PLoS Negl Trop Dis. 2021 Dec 8;15(12):e0009915. doi: 10.1371/journal.pntd.0009915 (PMC8654171; doi:10.1371/journal.pntd.0009915)
Supplement: S1 Table — No data for gender available for two communities (n = 102). (DOCX) [file pntd.0009915.s001.docx]

S1 Table: Estimated resident population (ERP) and numbers in survey for seven remote Central Australian communities, 2014-18

|  | **ERP** | **Tested** | **Total** | **p value** |
| --- | --- | --- | --- | --- |
| **Age** | **n (%)** | **n (%)** | **n (%)** |  |
| 5-14 years | 296 (19.26) | 124 (17.69) | 420 (18.77) | 0.296 |
| 15-24yrs | 301 (19.58) | 117 (16.55) | 417 (18.63) |  |
| 25-54yrs | 747 (48.6) | 373 (53.21) | 1,120 (50.04) |  |
| 55-64yrs | 117 (7.61) | 55 (7.85) | 172 (7.69) |  |
| 65 yrs and over | 76 (4.94) | 33 (4.71) | 109 (4.87) |  |
| Total | 1537 | 702 | 2239 |  |
| **Gender** |  |  |  |  |
| Male | 632 (47.8) | 288 (46.6) | 921 (47.5) | 0.61 |
| Female | 689 (52.2) | 330 (53.4) | 1019 (52.5) |  |
| Total | 1321 | 618 | 1939 |  |

**S1 Table.** Legend. Estimated resident population data not available for children aged 3-4 years (n=18). No data for gender available for two communities (n=102).
